# Targeting Phosphoinositide 3-Kinase to Reduce the Progression of Ovarian Cancer Cells in a 3D Collagen Model

**DOI:** 10.3390/biom16030377

**Published:** 2026-03-02

**Authors:** Alexandria B. Tino, Peter H. Sykes, Gabi U. Dachs, Kenny Chitcholtan

**Affiliations:** 1 Gynaecological Cancer Research Group, Department of Obstetrics and Gynaecology, University of Otago Christchurch, Christchurch 8011, New Zealand; alex.tino@otago.ac.nz (A.B.T.); peter.sykes@cdhb.health.nz (P.H.S.); kenny.chitcholtan@otago.ac.nz (K.C.); 2 Obstetrics & Gynaecology, Christchurch Women’s Hospital, Te Whatu Ora Waitaha—Health New Zealand, Christchurch 8140, New Zealand; 3 Mackenzie Cancer Research Group, Department of Pathology and Molecular Medicine, University of Otago Christchurch, Christchurch 8011, New Zealand

**Keywords:** ascites, TNF-α, LPA, cancer cell line, buparlisib, SN32976, pterostilbene

## Abstract

Ovarian cancer remains a major cause of mortality in women aged 74 years and under. Dysregulation of the PI3K/AKT/mTOR and NFκB signaling pathways has been associated with poor outcomes and treatment resistance. This study evaluated three potential anticancer agents targeting these pathways: buparlisib (a pan-PI3K/mTORC1 inhibitor), SN32976 (a PI3K p110α inhibitor), and pterostilbene (a resveratrol analogue that downregulates PI3K/AKT and NFκB signaling). Their efficacy was tested in 3D collagen models of ovarian cancer, using SKOV3 and OVCAR8 cell lines, activated by tumor necrosis factor-alpha (TNFα) and lysophosphatidic acid (LPA). Using concentrations derived from 2D assays, viability, collagen gel sizes, secretion of interleukin 6/8 (IL-6/8) and signal pathway proteins were analyzed. All compounds were less effective in 3D models than in 2D cultures, with high cell viability maintained. TNFα and LPA did not significantly alter drug sensitivity, and collagen gel contraction was largely unaffected. While the compounds did not consistently change signaling protein levels, they generally reduced secretion of pro-inflammatory cytokines IL-6 and IL-8. Growth in 3D collagen gels conferred drug resistance on OVCAR8 but not SKOV3 models. Overall, these findings provide preclinical support for further investigation of SN32976 and pterostilbene in ovarian cancer models.

## 1. Introduction

Despite declining incidence, ovarian cancer remains the leading cause of mortality from gynecological cancers in New Zealand, and second globally, in women aged 74 years and under [1,2,3]. The identification of novel agents to treat this disease is an active field in drug development [4].

In ovarian cancer, aberrations of the intracellular signaling cascades (phosphoinositide 3 kinase (PI3K)/protein kinase B (AKT)/mammalian target of rapamycin (mTOR) (PI3K/AKT/mTOR)) and nuclear factor kappa-light-chain-enhancer of activated B cells (NFκB) correlate with unfavorable patient outcomes and resistance to chemo-/radiotherapy [5]. There is considerable crosstalk between the PI3K/AKT/mTOR and NFκB pathways [6], and both pathways are highly mutated in ovarian cancer [6,7]. Despite limited clinical benefit observed with PI3K/AKT/mTOR inhibitors as monotherapy [8], interest in pathway inhibitors remains high [9,10], with better predictive models required.

Developed as a pan-PI3K inhibitor, buparlisib (NVP-BKM120) targets the class IA isoforms PI3Kα, PI3Kβ, and PI3Kδ; the class IB PI3Kγ; and mTORC1 [11]. Buparlisib has been investigated in combination with the poly(ADP-ribose) polymerase (PARP) inhibitor Olaparib, showing promise in ovarian cancer but with significant toxicities [12]. Buparlisib presents one of the most advanced pan-PI3K inhibitors in clinical development [13].

SN32976 was designed to increase the therapeutic window whilst limiting toxicity associated with pan-PI3K inhibitors, instead targeting the catalytic subunit PI3K p110α [14]. *PIK3CA* variants are one of the top three most common single nucleotide variants detected in ovarian tumors [15,16,17], allowing targeted therapy of about 10% of patients with ovarian cancer [17]. Despite promising preclinical data [14], SN32976 has not progressed into clinical testing, and its activity against ovarian cancer cells has not been assessed.

The phenolic compound pterostilbene is an analogue of resveratrol but with improved bioavailability [18,19]. A recent clinical trial of pterostilbene in endometrial cancer demonstrated good tolerability, but it was only effective at a transcriptomic level [20]. In vitro, pterostilbene downregulates PI3K/AKT [21,22] and NFκB signaling [23], showing activity against ovarian cancer cells [24].

Advanced ovarian cancer is characterized by tumor cells aggregating within abdominal ascitic fluid and embedding on distal organs [25]. Ascitic fluid is rich in cytokines, growth factors, and phospholipids essential for secondary tumor establishment and growth, including tumor necrosis factor-alpha (TNF-α) and lysophosphatidic acid (LPA) [25].

Most in vitro studies and initial drug screens rely on cell monolayers that poorly represent the tumor microenvironment [26,27]. More advanced 3D models were, therefore, designed to better represent clinical tumors [26,27]. A 3D collagen model was recently optimized to represent newly established ovarian cancer metastasis, activated by ascitic fluid constituents [28].

Our study was designed to test the efficacy of three potential anticancer compounds (buparlisib, SN32976, pterostilbene) in TNF-α- or LPA-activated 3D models of ovarian cancer.

## 2. Materials and Methods

### 2.1. Chemicals and Compounds

Chemicals were obtained from Sigma Aldrich (St. Louis, MA, USA), unless specified. Stocks of buparlisib and SN32976 (kindly provided by Associate Professor Stephen Jamieson, Faculty of Medical and Health Sciences, Pharmacology, University of Auckland, New Zealand [14]) and pterostilbene (Biotivia, New York, NY, USA) were dissolved in dimethyl sulfoxide.

### 2.2. Cell Lines and Maintenance

Human ovarian carcinoma cell lines SKOV3 (*PIK3CA* gain-of-function mutant, clear-cell serous ovarian cancer) and OVCAR8 (wild-type *PIK3CA*, high-grade serous ovarian cancer) were from the American Type Culture Collection (ATCC; Manassas, VA, USA) and authenticated by short tandem repeat (STR) testing (CellBank, Children’s Medical Research Institute, New South Wales, Australia). Cells were maintained in Dulbecco’s Modified Eagle Medium (DMEM) with 10% fetal bovine serum (FBS), PenStrep, GlutaMAX™ and Fungizone^®^ (Lab Supply, Dunedin, New Zealand) in a humidified 5% CO_2_ incubator.

### 2.3. Activated 3D Collagen Cell Model

Cells (2 × 10^5^ cells per 50 µL gel) were encapsulated in agarose with rat tail collagen I, and gels were grown in 2% DMEM for 6 days, as described before [28] (Supplementary Figure S1). Media was refreshed every 2 days. 3D cell models were activated by the addition of 20 μM LPA (Sapphire Bioscience, Sydney, NSW, Australia) or 20 nM TNF-α (Thermo Fisher Scientific, Auckland, New Zealand), as described before [28].

### 2.4. Dose Testing of 2D Cell Cultures

Cells grown in monolayers were exposed to 0.02–50 µM buparlisib or SN32976 or 0.2–2000 µM pterostilbene for 3 days. Cell response was tested using Alamar Blue (Thermo Fisher Scientific). The dose of compounds required to reduce metabolic activity by 50% (IC50) was determined from dose–response curves.

### 2.5. Dose Testing of 3D Models

After two days’ growth, the 2D IC50-equivalent concentration of buparlisib, SN32976 or pterostilbene was added to fresh media supplemented with LPA or TNF-α and grown for another four days. Controls contained the vehicles for the compounds (DMSO) and LPA (bovine serum albumin). At the six-day endpoint, cell viability (live–dead) was measured by staining with Calcein-AM and propidium iodide (Thermo Fisher Scientific), with six random regions of interest selected and counted per gel (Supplementary Figure S2). Gel size was measured by microscopy, and secretion of interleukin 8 (IL-8) and IL-6 was measured by enzyme-linked immunosorbent assay (ELISA) over the final 48 h (R&D Systems, Minneapolis, MN, USA), as previously described [28]. For each condition, at least three independent biological experiments were performed.

### 2.6. Western Blot Analysis

Signaling pathways of cells grown in 3D were measured by Western blot, using the method described in [29]. Primary and secondary antibodies and their dilutions are listed in Supplementary Table S1. Full size Western blots are shown in Supplementary Figures S3–S5.

### 2.7. Statistics

Data were graphed and statistically analyzed using GraphPad Prism (v10.3.1, Boston, MA, USA). Differences between two data sets were determined by Student’s *t*-test and, for >2 groups, by one-way ANOVA (significance * *p* < 0.05, ** *p* < 0.01, *** *p* < 0.001, **** *p* < 0.0001).

## 3. Results

### 3.1. Dose-Response of Monolayers to Experimental Compounds

**Figure 1:**
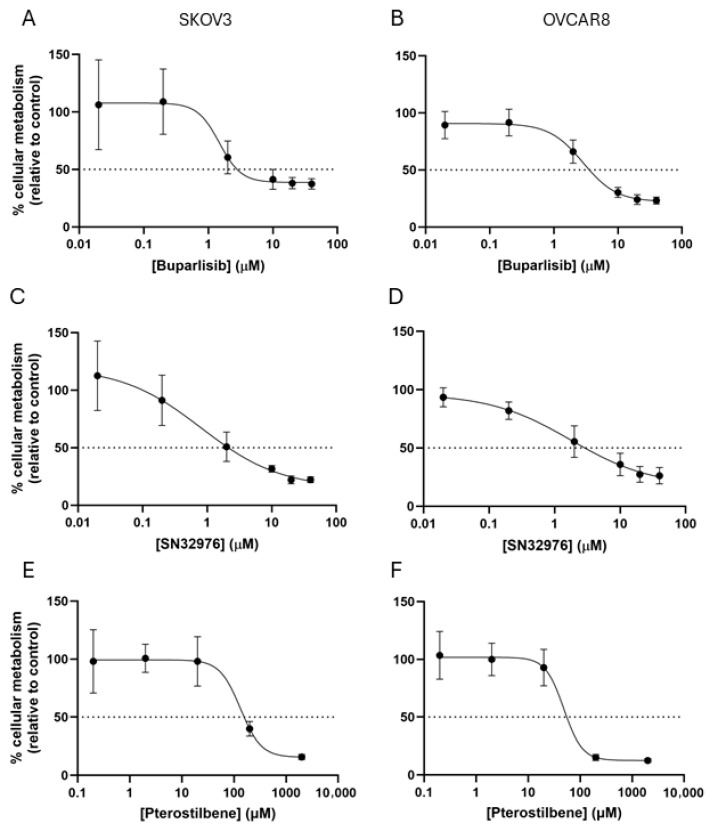
Treatment dose-response curves of monolayers of cells to three different compounds. Cells were treated with differing concentrations of buparlisib (**A**,**B**), SN32976 (**C**,**D**) or pterostilbene (**E**,**F**) for three days. Cellular metabolism was measured by the Alamar Blue assay. The dotted line represents a 50% inhibition of cellular metabolism. Data were fitted to a four-parameter, variable slope dose-response curve. Data represent ± SEM (*n* = 3).

The responses of the two ovarian cancer cell lines in a 2D model to the three compounds were assessed to determine the concentrations for subsequent 3D testing. SKOV3 appeared more sensitive than OVCAR8 to buparlisib and SN32976, whereas it appeared more resistant to pterostilbene (Figure 1). IC50 values for buparlisib were 1.5 and 2.5 μM, for SN32976 were 1.0 and 2.5 μM, and for pterostilbene were 130 and 50 μM for SKOV3 and OVCAR8, respectively. These 2D-derived IC50 concentrations were applied to 3D models in order to standardize across compounds and across cell lines.

### 3.2. Effect of Compounds on Cell Viability in Collagen 3D Models

**Figure 2:**
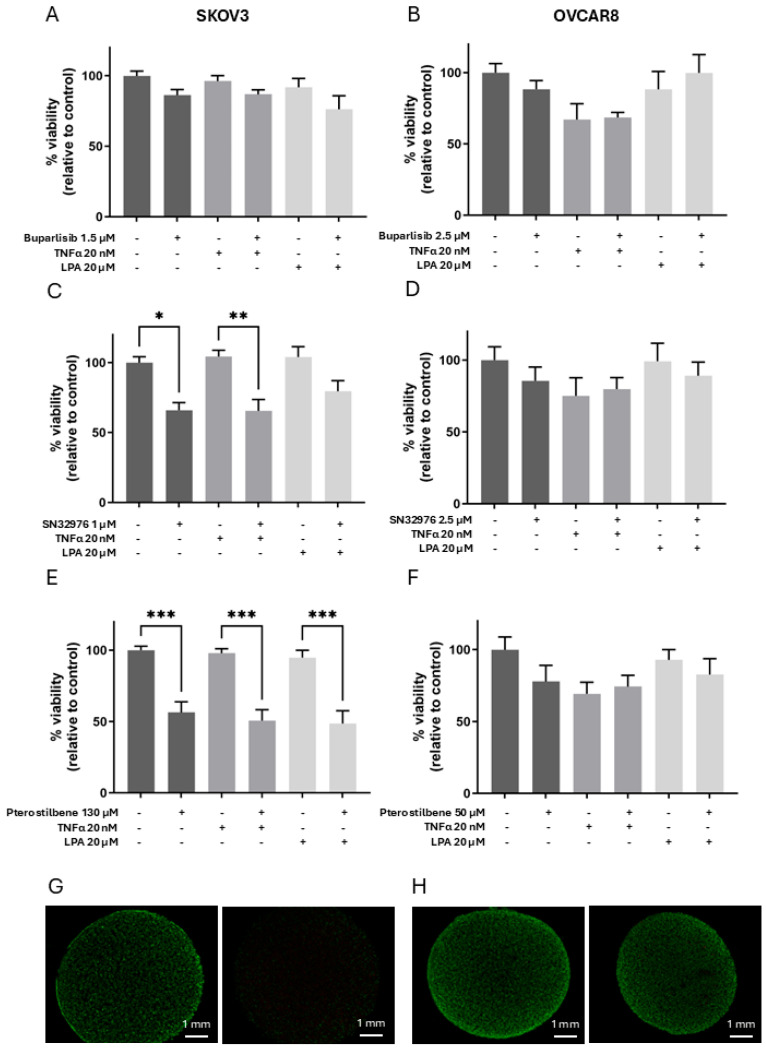
Viability of cells in 3D collagen in response to treatment with compounds alone or in the presence of either TNF-α or LPA. Analysis of live–dead cells of SKOV3 (**A**,**C**,**E**,**G**) and OVCAR8 (**B**,**D**,**F**,**H**), cell lines grown in collagen gels for 6 days. Cell-loaded gels were incubated for 2 days with only medium and then with a defined concentration of buparlisib (**A**,**B**), SN32976 (**C**,**D**), or pterostilbene (**E**–**H**), with TNF-α and/or LPA, and were incubated for a further 4 days. Gels were stained with calcein-AM and propidium iodide to detect live and dead cells, and micrographs were quantified using ImageJ (version 1.53d). Examples of fluorescent images are shown of SKOV3 (**G**) and OVCAR8 (**H**); the first image of each are control gels, and the second is treated with pterostilbene. Bars represent means + SEM (*n* = 3); dark-grey bars represent controls or compounds alone, mid-grey bars represent the addition of TNF-a (20 nM), and light-grey bars represent the addition of LPA (20 mM). Controls contained vehicle instead of compounds. Statistical significance * *p* < 0.05, ** *p* < 0.01, *** *p* < 0.001.

The IC50 concentrations of the three compounds, derived from the 2D compound-concentration curves, were utilised in experiments on the 3D models (Figure 2). To activate the models, TNFα and LPA were added during drug exposure at 20 nM and 20 µM, respectively.

In 3D models, buparlisib at 1.5 µM reduced the viability of SKOV3 cells by 14% and, at 2.5 µM, reduced the viability of OVCAR8 by 10% (Figure 2A,B). SN32976 at 1 µM and 2.5 µM, respectively, reduced viability of SKOV3 cells by 34% and OVCAR8 by 20% (Figure 2C,D). Pterostilbene at 130 µM and 50 µM, respectively, reduced viability of SKOV3 by 51% and OVCAR8 by 17% (Figure 2E,F).

The addition of TNFα or LPA to activate 3D models did not significantly alter the cell response to any of the three compounds (Figure 2).

### 3.3. Effect of Compounds on Collagen Gel Size

**Figure 3:**
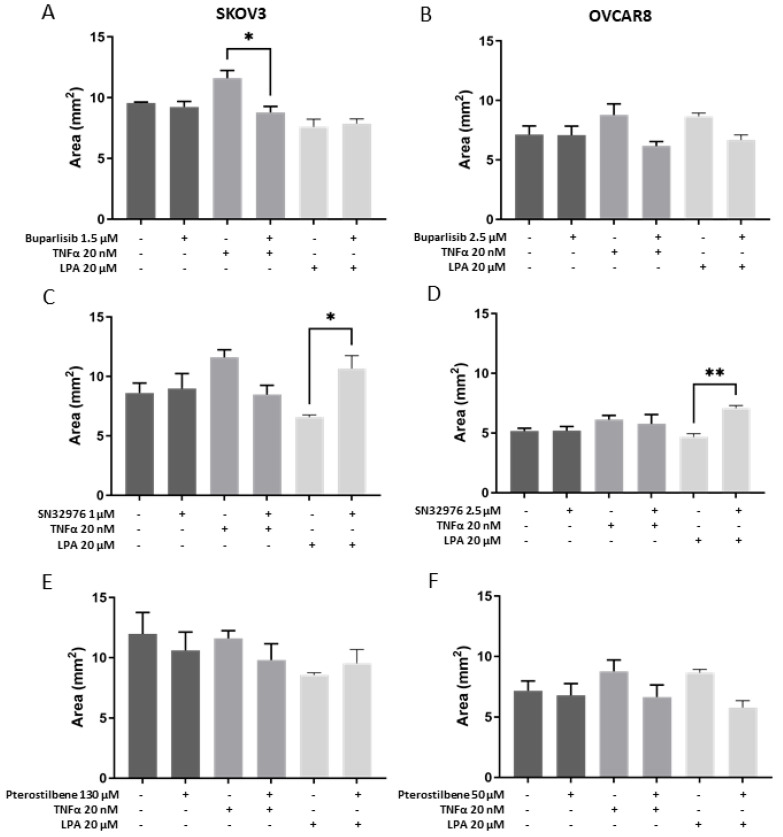
Size of collagen/cell matrices in response to treatment with compounds alone or in the presence of either TNF-α or LPA. Analysis of gel area of SKOV3 (**A**,**C**,**E**) and OVCAR8 (**B**,**D**,**F**) cell lines grown in collagen gels for 6 days. Cell-loaded gels (50 mL containing 2 × 10^5^ cells) were incubated for 2 days with only medium and then with vehicle (control gels) or a designated concentration of buparlisib, SN32976, or pterostilbene, with TNF-α or LPA added, and incubated for a further 4 days. Gel sizes were measured from micrographs using ImageJ. Bars represent means + SEM (*n* = 3 independent experiments); dark-grey bars represent controls or compounds alone, mid-grey bars represent the addition of TNF-a (20 nM), and light-grey bars represent the addition of LPA (20 mM). Statistical significance * *p* < 0.05, ** *p* < 0.01.

Gel sizes were measured as an indication of collagen remodeling, as determined previously over time and by collagen staining [28]. The addition of buparlisib, SN32976, or pterostilbene alone did not alter gel sizes relative to vehicle controls (Figure 3). In the SKOV3 model, buparlisib reduced gel sizes in the presence of TNF-α compared to TNF-α alone (Figure 3A), whereas SN32976 increased gel sizes in the presence of LPA compared to LPA alone (Figure 3C). In the OVCAR8 model, neither TNF-α nor LPA promoted collagen remodeling, and the addition of SN32976 to LPA resulted in larger gel sizes than LPA alone (Figure 3D). As in previous reports [28], gels containing OVCAR8 were notably smaller than those containing SKOV3 by day 6 (Figure 3).

### 3.4. Effect of Compounds on the Secretome

**Figure 4:**
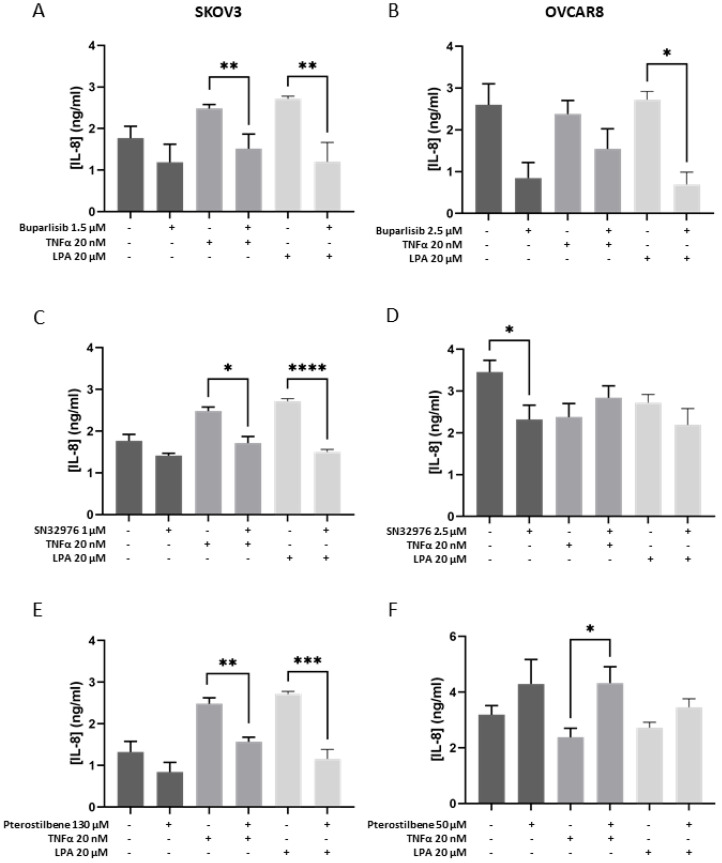
IL-8 secretion in response to treatment with compounds alone or in the presence of either TNF-α or LPA. The concentration of IL-8 was measured with ELISA in SKOV3 (**A**,**C**,**E**) and OVCAR8 (**B**,**D**,**F**) cell lines grown in collagen gels for 6 days. Cell-loaded gels were incubated for 2 days with only medium and then with vehicle (control gels) or a designated concentration of buparlisib, SN32976, or pterostilbene, with TNF-α or LPA added, and incubated for a further 4 days. Protein concentrations of IL-8 accumulated over 48 h in the culture medium are shown as mean + SEM from *n* = 3. Dark-grey bars represent controls or compounds alone, mid-grey bars represent the addition of TNF-a (20 nM), and light-grey bars represent the addition of LPA (20 mM). Statistical significance * *p* < 0.05, ** *p* < 0.01, *** *p* < 0.001, **** *p* < 0.0001.

**Figure 5:**
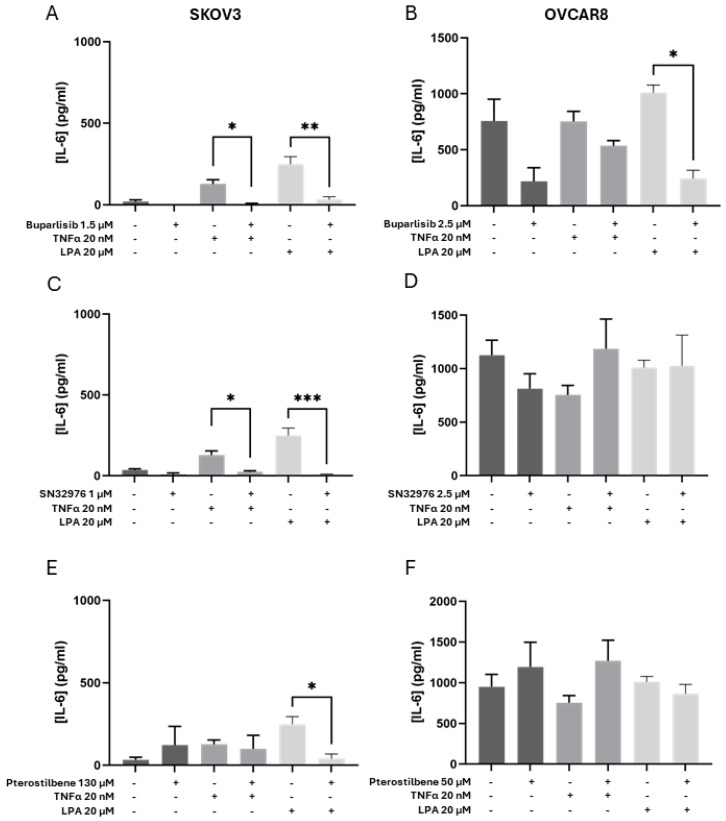
IL-6 secretion in response to treatment with compounds alone or in the presence of either TNF-α or LPA. The concentration of IL-6 was measured with ELISA in SKOV3 (**A**,**C**,**E**) and OVCAR8 (**B**,**D**,**F**) cell lines grown in collagen gels for 6 days. Cell-loaded gels were incubated for 2 days with only medium and then with vehicle (control gels) or a designated concentration of buparlisib, SN32976, or pterostilbene, with TNF-α and/or LPA added, and incubated for a further 4 days. Protein concentrations of IL-6 accumulated over 48 h in the culture medium are shown as mean + SEM from *n* = 3. Dark-grey bars represent controls or compounds alone, mid-grey bars represent the addition of TNF-a (20 nM), and light-grey bars represent the addition of LPA (20 mM). Statistical significance * *p* < 0.05, ** *p* < 0.01, *** *p* < 0.001.

Secretion of IL-8 and IL-6 was measured as a marker of the compounds’ ability to modify the promotion of angiogenesis, tumor cell survival and expansion. Buparlisib alone tended to reduce IL-8 and IL-6 secretion in both cell line models compared with controls (Figure 4A,B and Figure 5A,B). SN32976 alone significantly reduced IL-8 secretion in the OVCAR8 model (Figure 4D). Pterostilbene alone did not affect cytokine secretion (Figure 4 and Figure 5).

The addition of TNF-α or LPA to the SKOV3 model increased the secretion of IL-8 and IL-6, whereas secretion of IL-8 and IL-6 from the OVCAR8 model appeared unaffected (as reported in [28]) (Figure 4 and Figure 5).

Buparlisib tended to reduce the TNF-α-increased secretion of IL-8 and IL-6 in both cell lines (Figure 4A,B and Figure 5A,B, respectively). Buparlisib was able to significantly reduce the LPA-increased secretion of IL-8 and IL-6 in SKOV3 to control levels (Figure 4A and Figure 5A,B) and of IL-8 for OVCAR8 (Figure 4B).

SN32976 significantly reduced the TNF-α- and LPA-induced secretion of IL-8 and IL-6 from SKOV3 to control levels (Figure 4C and Figure 5C). SN32976 had a limited effect on activated OVCAR8 models.

Pterostilbene was able to reduce the LPA-induced secretion of IL-8 and IL-6 in the SKOV3 model (Figure 4E and Figure 5E). In the activated OVCAR8 model, TNF-α suppressed IL-8 secretion, and pterostilbene antagonised this suppression (Figure 4F).

### 3.5. Cell Signaling Pathways in 3D Ovarian Cancer Models

**Figure 6:**
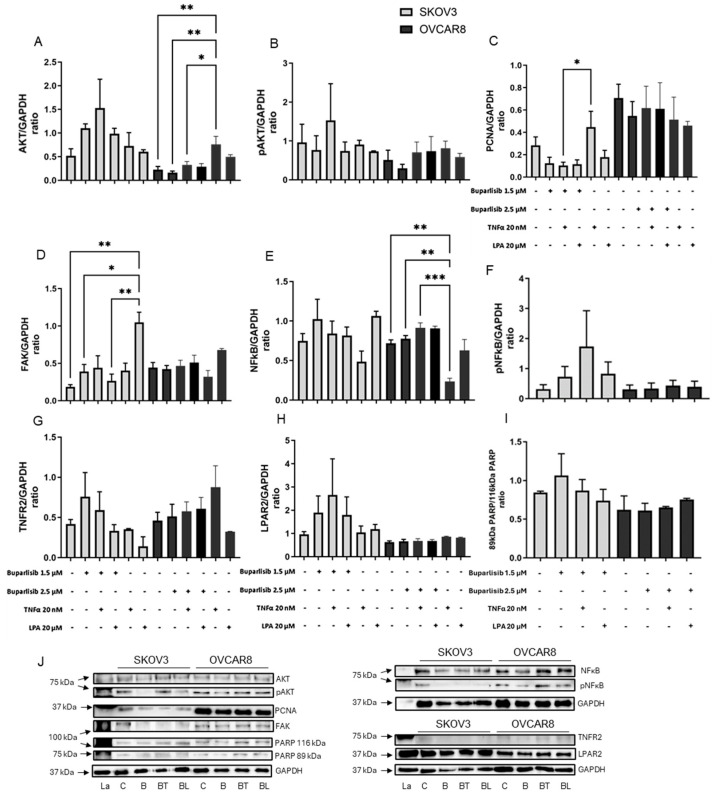
Impact of buparlisib, alone or with TNF-α or LPA, on signal protein expression. SKOV3 and OVCAR8 cell models were grown for a total of 6 days. Cell-loaded gels were incubated for 2 days with only medium and then with vehicle (control gels) or buparlisib (1.5 µM for SKOV3 and 2.5 µM for OVCAR8), 20 nM TNF-α, and/or 20 µM LPA, and incubated for a further 4 days. Bars represent means + SEM (*n* = 3) protein expression normalized to GAPDH, showing AKT (**A**), pAKT (**B**), PCNA (**C**), FAK (**D**), NFκB (**E**), pNFκB (**F**), TNFR2 (**G**), LPAR2 (**H**), and uncleaved and cleaved PARP (**I**). Examples of Western blots are shown (**J**). Statistical significance * *p* < 0.05, ** *p* < 0.01, *** *p* < 0.001. La—ladder, C—control, T—TNF-α, L—LPA, B—buparlisib, BT—buparlisib and TNF-α, BL—buparlisib and LPA. The original Western blots are shown in Figure S3 of the Supplementary Materials.

**Figure 7:**
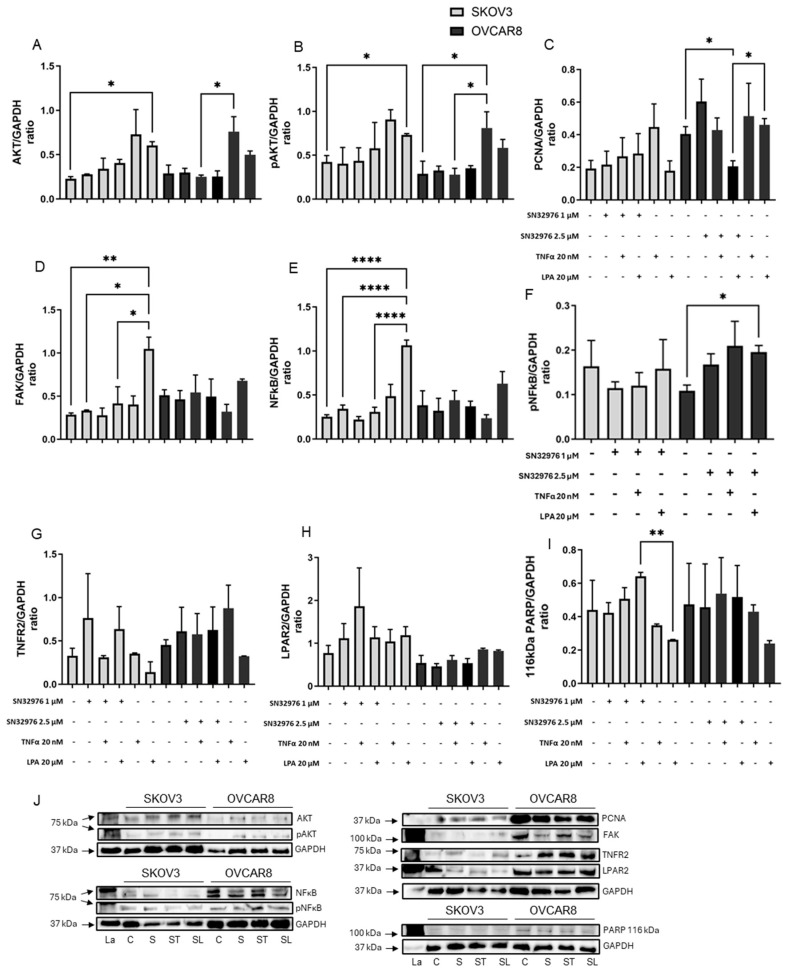
Impact of SN32976, alone or with TNF-α or LPA, on signal protein expression. SKOV3 and OVCAR8 cell models were grown for a total of 6 days. Cell-loaded gels were incubated for 2 days with only medium and then with vehicle (control gels) or SN32976 (1.0 µM for SKOV3 and 2.5 µM for OVCAR8), 20 nM TNF-α, and/or 20 µM LPA, and incubated for a further 4 days. Bars represent means + SEM (*n* = 3) protein expression normalized to GAPDH, showing AKT (**A**), pAKT (**B**), PCNA (**C**), FAK (**D**), NFκB (**E**), pNFκB (**F**), TNFR2 (**G**), LPAR2 (**H**), and uncleaved PARP (**I**). Examples of Western blots are shown (**J**). Statistical significance * *p* < 0.05, ** *p* < 0.01, **** *p* < 0.0001. La—ladder, C—control, T—TNF-α, L—LPA, S—SN32976, ST—SN32976 and TNF-α, SL—SN32976 and LPA. The original Western blots are shown in Figure S4 of the Supplementary Materials.

**Figure 8:**
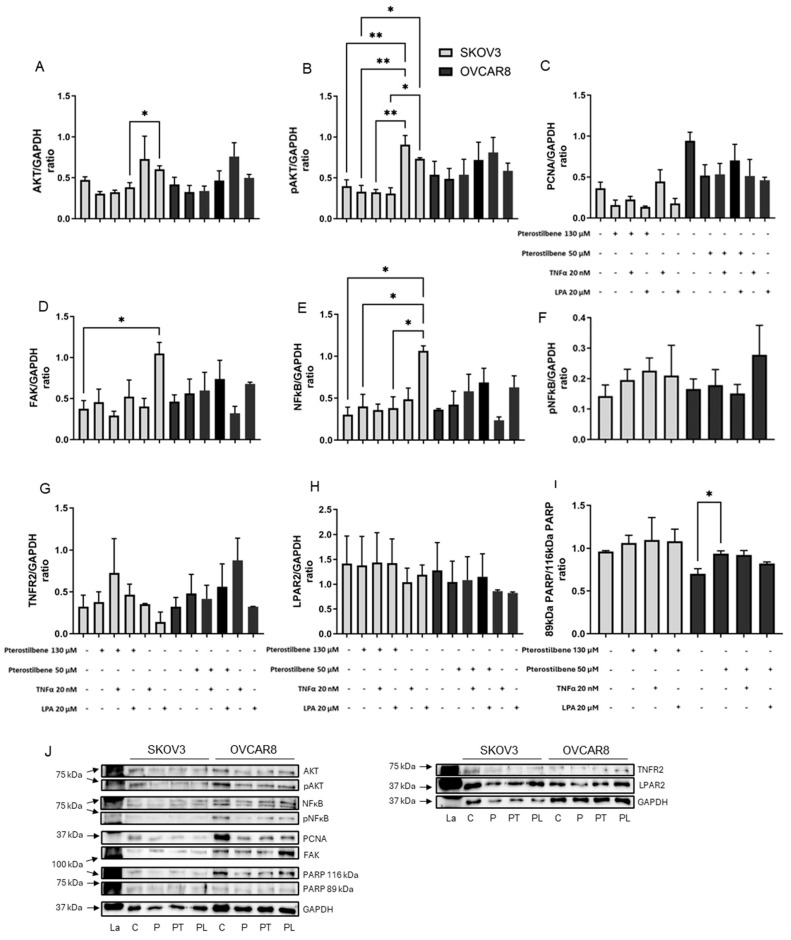
Impact of pterostilbene, alone or with TNF-α or LPA, on signal protein expression. SKOV3 and OVCAR8 cell models were grown for a total of 6 days. Cell-loaded gels were incubated for 2 days with only media and then vehicle (control gels) or pterostilbene (130 µM for SKOV3 and 50 µM for OVCAR8), 20 nM TNF-α, and/or 20 µM LPA were added, and gels were incubated for a further 4 days. Bars represent means + SEM (*n* = 3) protein expression normalized to GAPDH, showing AKT (**A**), pAKT (**B**), PCNA (**C**), FAK (**D**), NFκB (**E**), pNFκB (**F**), TNFR2 (**G**), LPAR2 (**H**), and uncleaved and cleaved PARP (**I**). Examples of Western blots are shown (**J**). Statistical significance * *p* < 0.05, ** *p* < 0.01. La—ladder, C—control, T—TNF-α, L—LPA, P—pterostilbene, PT—pterostilbene and TNF-α, PL—pterostilbene and LPA. The original Western blots are shown in Figure S5 of the Supplementary Materials.

The effects of TNF-α, LPA and the three compounds on PI3K signaling pathways were measured by Western blot (Figure 6, Figure 7 and Figure 8). Total expression and phosphorylation of AKT, an integral protein in the PI3K signaling pathway, were determined. Total expression and phosphorylation of NFκB and total expression of FAK were measured as indicators of possible effects on crosstalk. PCNA was measured as an indicator of the effect on cell proliferation. The total expression of the TNF-α target receptor TNFR2 and the LPA target receptor LPAR2 was also measured. Uncleaved PARP vs. cleaved PARP were measured as an indicator of apoptosis. GAPDH, used as an internal control, was unaffected by the treatments.

Overall, TNF-α tended to increase PCNA in SKOV3 and increase total AKT and decrease NFκB in OVCAR8 (Figure 6A,C,E, Figure 7A,C,E and Figure 8A,C,E). LPA tended to increase AKT, pAKT, FAK and NFκB in SKOV3 (Figure 6A,B,D,E, Figure 7A,B,D,E and Figure 8A,B,D,E). Following exposure to ascites components alone, pNFκB and cleaved PARP could not be detected in either cell line. The receptors TNFR2 and LPAR2 were present but unaffected by the ascites constituents.

None of the compounds alone notably affected cell signaling pathway proteins or markers of proliferation or apoptosis (Figure 6, Figure 7 and Figure 8). However, the activation by TNF-α and LPA affected the response of the models to the compounds.

Buparlisib tended to reverse the TNF-α- or LPA-induced modifications in protein levels. In OVCAR8, buparlisib reversed TNF-α- induced increases in AKT and decreases in NF-κB (Figure 6A and Figure 6E, respectively). In SKOV3, buparlisib suppressed LPA-induced expression of FAK (Figure 6D) and TNF-α-induced PCNA expression (Figure 6C). Buparlisib did not affect levels of TNFR2 and LPAR2 (Figure 6G,H). Apoptosis marker changes were not observed at the tested buparlisib concentrations (Figure 6I).

SN32976 similarly reversed some of the effects induced by ascites constituents (Figure 7). SN32976 repressed the effect of LPA in SKOV3, reducing AKT, pAKT, FAK and NFκB to control levels (Figure 7A,B,D,E). SN32976 increased expression of LPA-induced PARP levels (Figure 7I), but cleaved PARP could not be quantified. In OVCAR8, SN32976 reduced TNF-α-induced expression of AKT and pAKT to control levels (Figure 7A,B). The combination of SN32976 and LPA reduced PCNA expression (Figure 7C).

Pterostilbene reversed LPA-induced expression of all signaling proteins measured in SKOV3: AKT, pAKT, FAK and NFκB (Figure 8A,B,D,E). In OVCAR8, pterostilbene increased cleaved PARP (Figure 8I). PCNA and the receptors TNFR2 and LPAR2 appeared unaffected by pterostilbene in both models (Figure 8C,G,H).

## 4. Discussion

This study aimed to elucidate the effect of three potential drugs in a 3D collagen gel model of ovarian cancer to assess their suitability for further development. The pan-class I PI3K inhibitor buparlisib did not reduce viability or collagen remodeling and had a modest effect on IL-8 and IL-6 secretion in both cell lines. In comparison, the specific PI3Kα-preferential inhibitor SN23976 was more effective in reducing SKOV3’s malignant behavior induced by LPA but had little impact on OVCAR8. The natural compound pterostilbene was effective in reducing LPA-induced malignant behavior in SKOV3 but had little effect on OVCAR8. There was no synergism evident between TNF-α and either of the three compounds. Collectively, these findings indicate that OVCAR8 cells exhibited reduced sensitivity to all three compounds within the 3D collagen I environment. While direct comparative experiments isolating the specific contribution of collagen I were not performed, the observed differential response suggests that microenvironmental factors associated with the collagen-based 3D architecture may contribute to reduced drug responsiveness.

In the present study, IC50 values derived from 2D monolayer cultures were applied to the 3D collagen model as standardized reference concentrations to enable direct comparison between culture systems. Although it is established that 3D models often exhibit increased drug resistance due to enhanced cell–cell and cell–matrix interactions, diffusion gradients, and microenvironment-driven transcriptional adaptations, using 2D-derived IC50 values allowed us to specifically assess the magnitude of microenvironment-mediated modulation of drug response. The reduced sensitivity observed in the 3D model highlights the biological relevance of spatial architecture and supports the notion that conventional 2D assays may underestimate therapeutic resistance. Relative IC50 concentrations were used to estimate the effectiveness of compound exposure in different cell models. Both Alamar Blue and calcein-AM measure cellular metabolic function and indirectly assess cell viability [30]. However, in a 3D setting, calcein-AM appeared to be more reliable than Alamar Blue [31,32]. The effectiveness of the compounds was reduced in the 3D model compared with the 2D model, consistent with previous reports using different drugs [33,34]. However, there was a distinct difference between cell lines.

In preclinical models, buparlisib demonstrated activity, as evidenced by in vitro growth restriction and xenograft tumor inhibition [11,35]. However, in clinical trials, buparlisib was a poor performer, with clinical benefit rates ranging from 8% to 23% and significant adverse effects [36,37,38,39].

Buparlisib 2D IC50 concentrations are biologically relevant (peak plasma concentration ~1.2 μM [40]). Our study showed that SKOV3 was slightly more sensitive to buparlisib than OVCAR8 in 2D (1.5 vs. 2.5 mM), which was unexpected as SKOV3 carries a *PIK3CA* activating mutation [37] and OVCAR8 is wild-type *PIK3CA* [41]. The 2D IC50 concentration of buparlisib was ineffective at reducing collagen remodeling or cell viability in the 3D models, which may indicate low gel penetrance. Gel penetrance was not tested directly but appears unlikely, as the compound was able to significantly modify the secretome in TNF-a- and LPA-activated 3D models.

Buparlisib reduced LPA-induced IL-8 and IL-6 secretion in both cell models. In breast cancer, IL-8 is a biomarker of resistance and metastasis, and an increased concentration of IL-8 was correlated with a higher level of AKT [42]. IL-8 and IL-6 are also prospective biomarkers of poor prognosis in ovarian cancer [43]. The secretion of IL-8 and IL-6 by ovarian cancer has been linked to FAK/PI3K signaling [44,45].

The effect of buparlisib on signaling pathways was subtle. The compound reduced levels of the motility signaling factor FAK, which is associated with ovarian cancer cell survival and platinum chemotherapy resistance [46,47]. A reduction in AKT signaling was only observed in TNF-α-activated OVCAR8 models. This could suggest activation of alternative signaling pathways. There is evidence in *PIK3CA*-mutated breast and cervical cancers that an alternative pathway involves serum- and glucocorticoid-regulated kinases, which can be activated by PDK1, bypassing AKT involvement and reducing dependence on PI3K signaling [48,49]. Alternatively, the presence of collagen I in 3D may have activated integrin signaling independent of PI3K signaling, which is linked to cancer cell proliferation and migration [50,51].

Preclinical data for SN32976 are scarce, and it was never tested against ovarian cancer in vitro, in vivo or clinically. The 2D IC50 concentration of SN32976 was similar to that of buparlisib, but in 3D, it was more effective than buparlisib. This supports the more favorable in vivo response compared with pan-PI3K inhibitors observed by Rewcastle et al. [14]. The sensitivity of SKOV3 to SN32976 was expected due to its gain-of-function *PIK3CA* mutation [52] and suggests that SN32976 may be a potential candidate to target clear-cell ovarian carcinoma. SN32976 reduced viability and IL-8 and IL-6 secretion when compared to LPA-activated models. SN32976 thus warrants further investigation in ovarian cancer cell lines and models.

Significantly higher concentrations of pterostilbene than either buparlisib or SN32976 were required to reduce cell viability in 2D and show an effect in 3D (20–130-fold higher). This confirms published data from monolayer cultures on pterostilbene [53,54]. The IC50 concentrations are safe for human use [23]. Compared with PI3K inhibitors, pterostilbene in 2D was more effective against OVCAR8 than against SKOV3. However, in 3D, SKOV3 was again more susceptible to the compound than OVCAR8, supporting the notion that it may be a useful adjuvant in clear-cell ovarian carcinoma [54]. Pterostilbene targets PI3K, NFκB, and FAK signaling pathways and thus may be most effective in combination with a JAK/Stat3 inhibitor such as ruxolitinib [55] or an ERK inhibitor such as SCH772984 [56].

Epithelial ovarian cancers are classified into five principal histotypes of high-grade serous, low-grade serous, mucinous, endometrioid and clear-cell carcinoma [57]. In this study, we chose an example each of clear-cell and high-grade serous ovarian carcinoma, based on published data (ATCC). However, how representative these cell lines are of their respective histological subtypes remains debatable [58,59]. Our previous study in this 3D model showed that SKOV3 exhibited fibroblastic morphology and modified the collagen I scaffold, resembling clinical samples of high-grade serous ovarian cancer [28] and not clear-cell ovarian cancer as purported. In comparison, OVCAR8 was migratory and developed collagen I channels, similar to those observed in clinical samples of clear-cell ovarian cancer [28] and unlike high-grade serous carcinoma.

This study had several limitations. Although the biological relevance of 3D models for drug testing had been established [26,27], they do not recapitulate the in vivo complexity of an organism. While our activated 3D model has considered the effect of matrix and ascites components [28], stromal components, such as fibroblast, endothelial or immune cells, were not incorporated. These may increase drug resistance directly or indirectly in a clinical setting [60]. Only two cell lines were tested, and further investigations are required to confirm their relevance to specific clinical histotypes. For signaling pathway analysis, cells in 3D grow more slowly than in 2D, resulting in lower protein yield [61]. 3D cultures have been found to have decreased phosphorylation compared to 2D, making it more challenging to detect by Western blot [61].

## 5. Conclusions

Our study on complex 3D models contributes valuable preclinical data on the response of ovarian cancer cell lines to buparlisib, SN32976 and pterostilbene. Our systematic evaluation highlights the unique responses of each cell line to collagen, ascites constituents and compounds, indicating that SN32976 and pterostilbene warrant further investigation in ovarian cancer models.

## Data Availability

All generated data and their analyses are shown in the article. Reasonable requests can be directed to the corresponding authors.

## Supplementary Materials

The following supporting information can be downloaded at: https://www.mdpi.com/article/10.3390/biom16030377/s1, Table S1: Antibodies used for Western blotting. Figure S1. Representative images of collagen/cell matrices. Figure S2. Representative fluorescent images of regions of interest for viability analysis of SKOV3 and OVCAR8 grown for 6 days. Figures S3–S5: Full size Western blots for Figure 6, Figure 7 and Figure 8, respectively.

## Abbreviations

The following abbreviations are used in this manuscript:

3D	three dimensional
AKT	protein kinase B
ELISA	enzyme-linked immunosorbent assay
FAK	focal adhesion kinase
GAPDH	glyceraldehyde-3-phosphate dehydrogenase
IC50	concentration required to reduce metabolic activity by 50%
IL6, IL8	interleukin 6, 8
LPA	lysophosphatidic acid
LPAR2	LPA receptor 2
mTOR	mammalian target of rapamycin
NFκB	nuclear factor kappa-light-chain-enhancer of activated B cells
PARP	poly(ADP-ribose) polymerase
PCNA	proliferating cell nuclear antigen
PI3K	phosphoinositide 3 kinase
TNF-α	tumor necrosis factor-alpha
TNFR2	TNF receptor 2
