# Supplementary material for: Targeting Phosphoinositide 3-Kinase to Reduce the Progression of Ovarian Cancer Cells in a 3D Collagen Model
Source: Biomolecules. 2026 Mar 2;16(3):377. doi: 10.3390/biom16030377 (PMC13024705; doi:10.3390/biom16030377)
Supplement: Supplementary file 1 [file biomolecules-16-00377-s001.zip › biomolecules-4136554-supplementary.pdf]

Supplementary materials

Table S1. Antibodies used for Western blotting.

Primary Antibody	Source, Dilution	Company (Product Code)	Secondary Antibody	Company (Product Code)
NF-κB p65 (65 kDa)	Polyclonal rabbit, 1:500	Santa Cruz Biotechnology (sc-372)	Polyclonal goat anti-rabbit HRP, 1:10,000	Santa Cruz Biotechnology (sc-2004)
p-NF-κB p65 (65 kDa)	Polyclonal rabbit, 1:500	Santa Cruz Biotechnology (sc-33020)	Polyclonal goat anti-rabbit HRP, 1:10,000	Santa Cruz Biotechnology (sc-2004)
Akt-1/2/3 (62 kDa)	Polyclonal rabbit, 1:1000	Santa Cruz Biotechnology (sc-8312)	Polyclonal goat anti-rabbit HRP, 1:10,000	Santa Cruz Biotechnology (sc-2004)
p-Akt-1/2/3 (62 kDa)	Polyclonal rabbit, 1:1000	Santa Cruz Biotechnology (sc-7985)	Polyclonal goat anti-rabbit HRP, 1:10,000	Santa Cruz Biotechnology (sc-2004)
TNFR2 (75 kDa)	Monoclonal mouse, 1:500	Santa Cruz Biotechnology (sc-393614)	Polyclonal donkey anti-mouse HRP, 1:10,000	Santa Cruz Biotechnology (sc-2314)
LPAR2 (39 kDa)	Polyclonal rabbit, 1:500	Abcam (ab135980)	Polyclonal goat anti-rabbit HRP, 1:10,000	Santa Cruz Biotechnology (sc-2004)
PCNA (36 kDa)	Monoclonal mouse, 1:500	Santa Cruz Biotechnology (sc-25280)	Polyclonal donkey anti-mouse HRP, 1:10,000	Santa Cruz Biotechnology (sc-2314)
PARP-1 (89 kDa, 116 kDa)	Polyclonal rabbit, 1:500	Santa Cruz Biotechnology (sc-7150)	Polyclonal goat anti-rabbit HRP, 1:10,000	Santa Cruz Biotechnology (sc-2004)
FAK (125 kDa)	Polyclonal rabbit, 1:500	Santa Cruz Biotechnology (sc-558)	Polyclonal goat anti-rabbit HRP, 1:10,000	Santa Cruz Biotechnology (sc-2004)
GAPDH (36 kDa)	Polyclonal rabbit, 1:10,000	Sigma-Aldrich (G9545)	Polyclonal goat anti-rabbit HRP, 1:10,000	Santa Cruz Biotechnology (sc-2314)

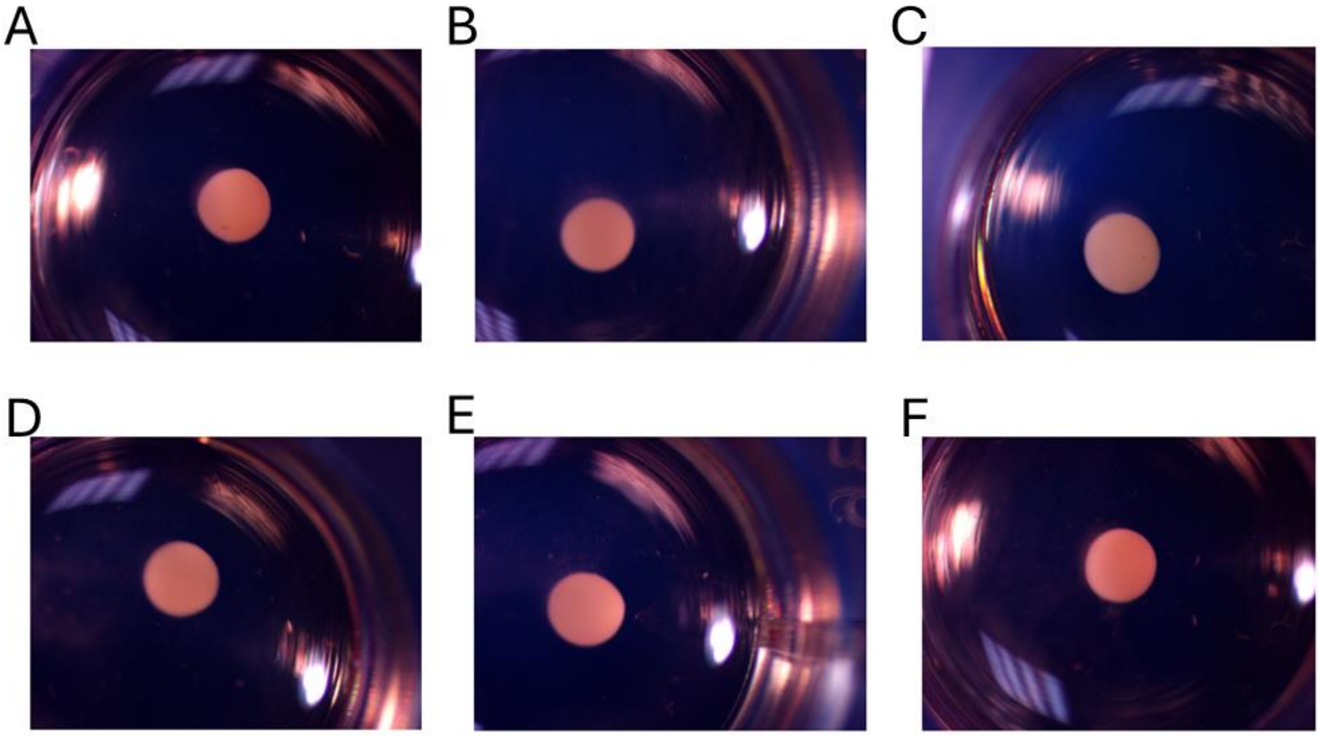

Figure S1. Representative images of collagen/cell matrices. Examples are shown for SKOV3 cells encapsulated in gel and grown for 6 days. Control gel (**A**), 1.5 μM buparlisib added (**B**), 20 nM TNFα added (**C**), 1.5 μM buparlisib + 20 nM TNFα added (**D**), 20 μM LPA added (**E**), and 1.5 μM buparlisib + 20 μM LPA added (**F**).

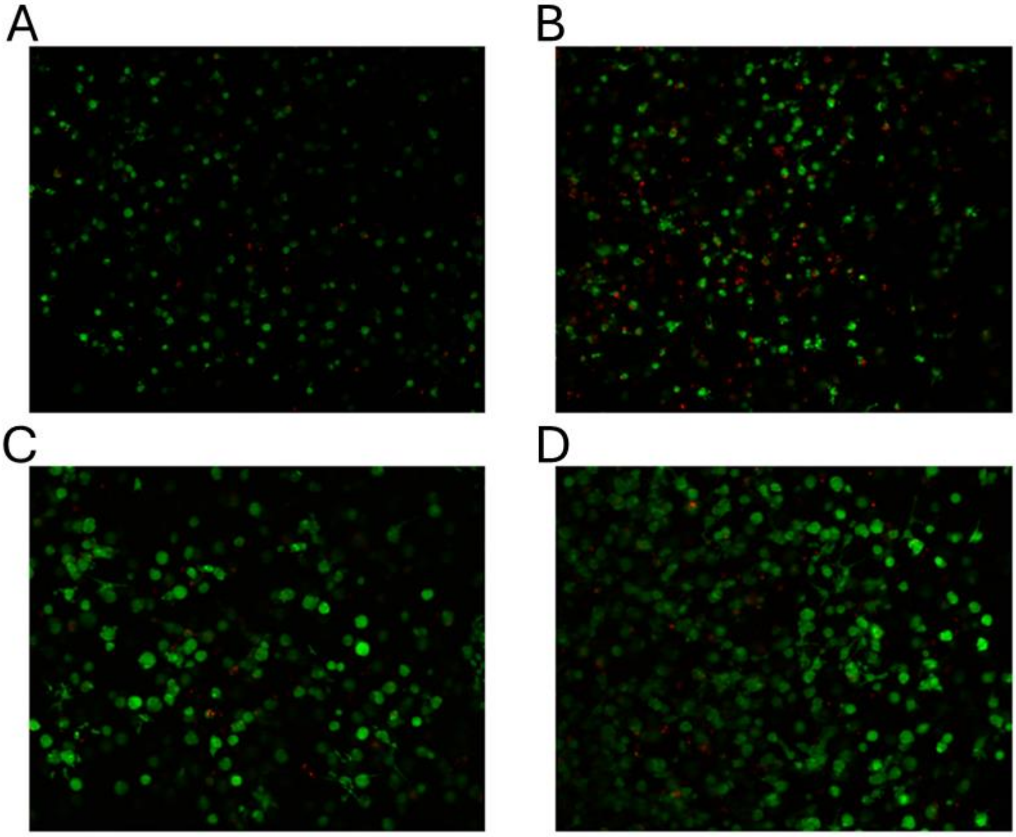

Figure S2. Representative fluorescent images of regions of interest for viability analysis of SKOV3 (**A**,**B**) and OVCAR8 (**C**,**D**) grown for 6 days. Control (**A**,**C**), 1 μM SN32976 added (**B**) or 2.5 μM

SN32976 added (**D**). Gels were stained with calcein-AM and propidium iodide to detect live (green) and dead (red) cells.

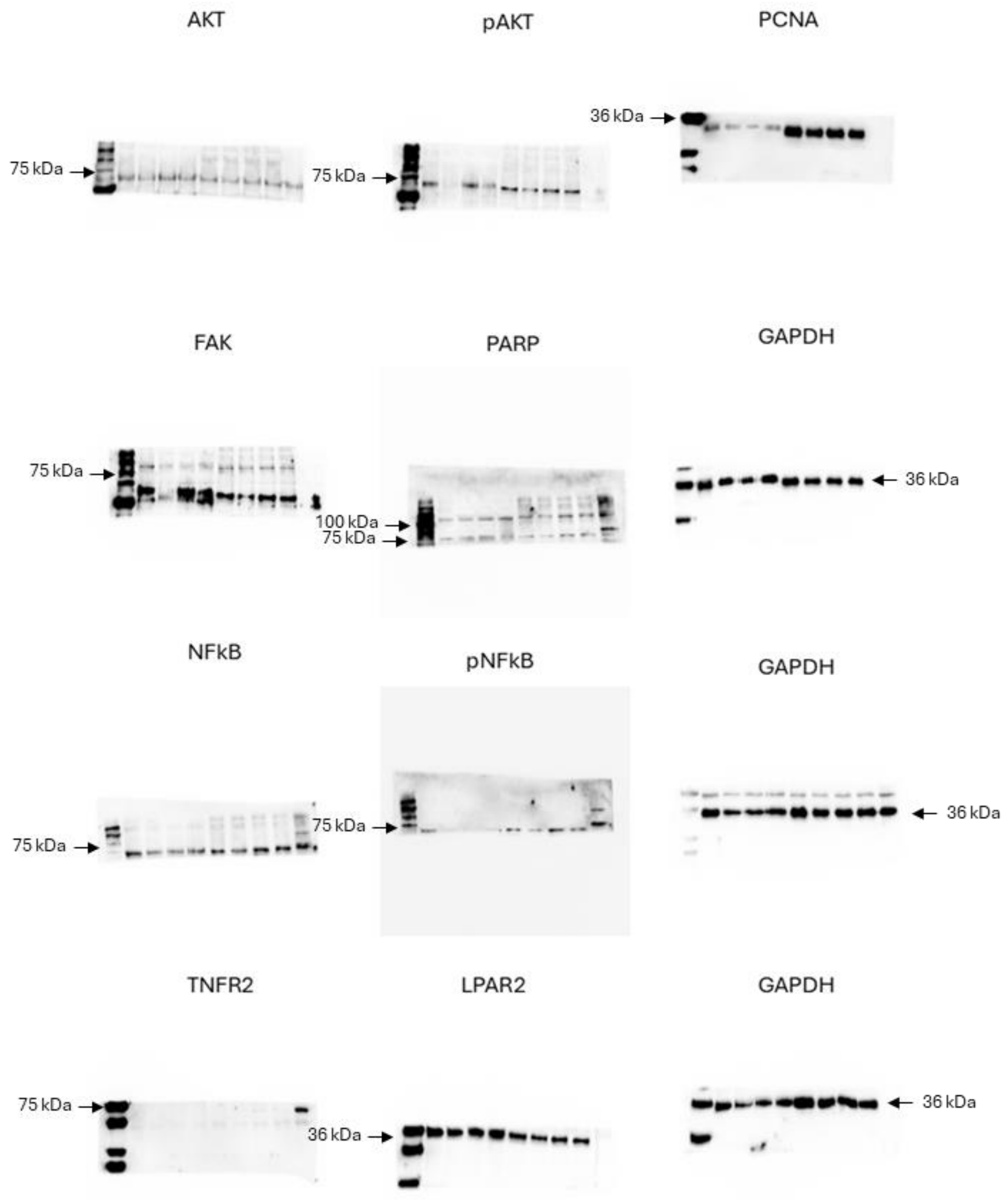

Figure S3. Uncropped images of full Western blots of Figure 6.

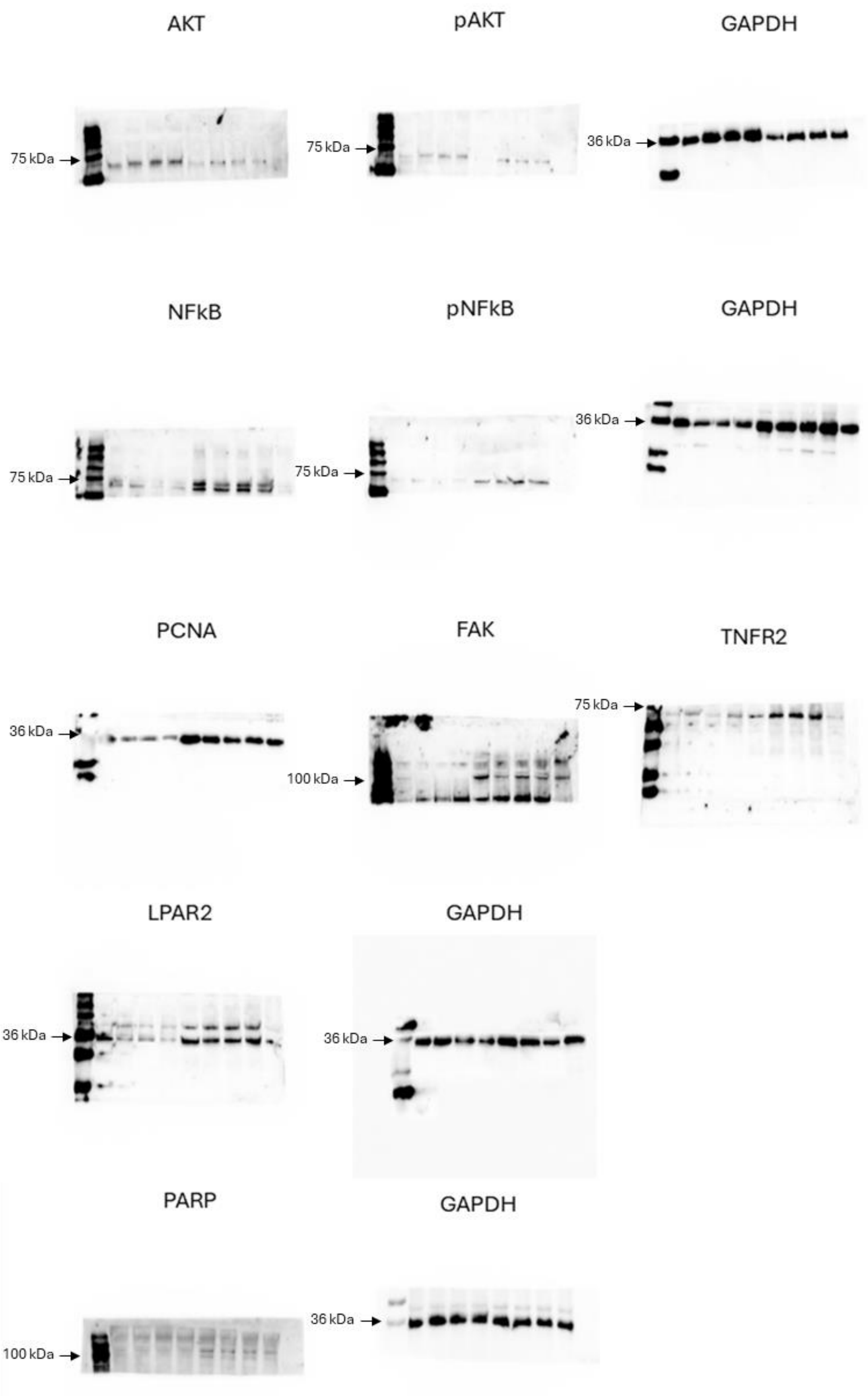

Figure S4. Uncropped images of full Western blots of Figure 7.

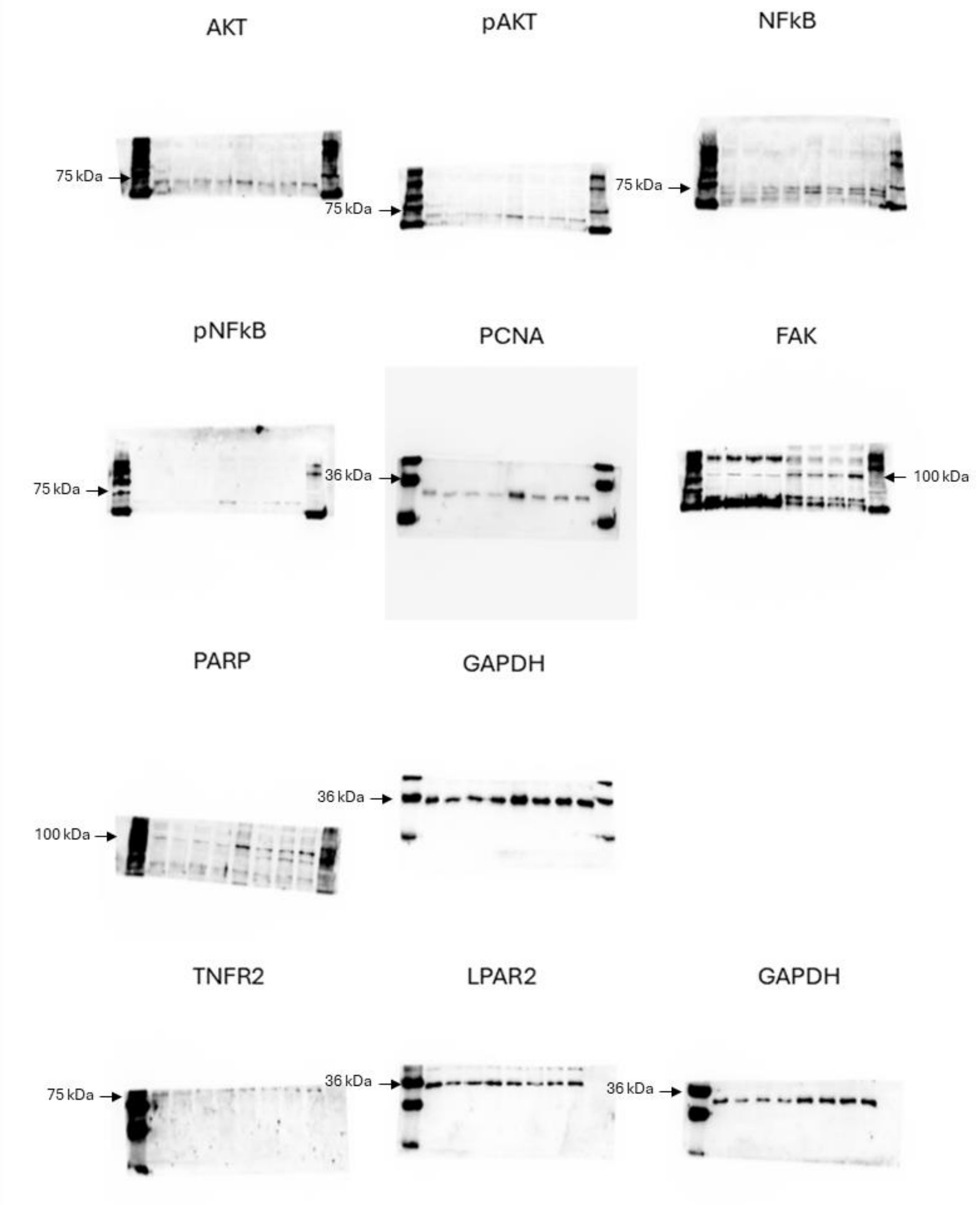

Figure S5. Uncropped images of full Western blots of Figure 8.
